# Study of Resource Allocation for 5G URLLC/eMBB-Oriented Power Hybrid Service

**DOI:** 10.3390/s23083884

**Published:** 2023-04-11

**Authors:** Huan Xie, Qiuming Zhang, Shu Du, Yang Yang, Xue Wu, Peng Qin, Runze Wu, Xiongwen Zhao

**Affiliations:** 1 State Grid Sichuan Electric Power Company, Information Communication Company, Chengdu 610041, China; 2 School of Electrical and Electronic Engineering, North China Electric Power University, Beijing 102206, China

**Keywords:** enhanced mobile broadband (eMBB) service, ultra-reliable low-latency communication (URLLC) service, power Internet of Things, resource allocation

## Abstract

With the rapid development of the 5G power Internet of Things (IoT), new power systems have higher requirements for data transmission rates, latency, reliability, and energy efficiency. Specifically, the hybrid service of enhanced mobile broadband (eMBB) and ultra-reliable low-latency communication (URLLC) has brought new challenges to the differentiated service of the 5G power IoT. To solve the above problems, this paper first constructs a power IoT model based on NOMA for the mixed service of URLLC and eMBB. Considering the shortage of resource utilization in eMBB and URLLC hybrid power service scenarios, the problem of maximizing system throughput through joint channel selection and power allocation is proposed. The channel selection algorithm based on matching as well as the power allocation algorithm based on water injection are developed to tackle the problem. Both theoretical analysis and experimental simulation verify that our method has superior performance in system throughput and spectrum efficiency.

## 1. Introduction

With the rapid construction of the power Internet of Things (IoT) based on 5G technology, there are new differentiated power system business requirements such as power consumption information collection, power transmission and transformation status detection, and accurate load control. For meeting the higher requirements of the above services for high data rate, low latency, reliability, energy efficiency, etc., the International Telecommunication Union (ITU) has proposed three types of services: massive machine type communication (mMTC), enhanced mobile broadband (eMBB), and ultra-reliable low-latency communication (URLLC) [1]. In particular, the URLLC technology-based power IoT is able to transmit electricity consumption information quickly [2,3]; eMBB technology is able to enhance and maintain the wireless network rate in a high and stable state. The coexistence of different service categories within the same time/frequency resources, especially eMBB and URLLC services, brings new challenges to differentiated services in 5G power networks [2,3,4].

In recent years, eMBB and URLLC hybrid services have received much attention [5,6,7,8]. In the Literature [5], a system model consisting of a base station (BS) and a reconfigurable intelligent surface (RIS) was proposed to study the coexistence of eMBB and URLLC services in cellular networks with the assistance of RIS. Literature [6] considered the low delay requirement and the shortage of spectrum resources of URLLC, so a method of multiplexing eMBB and URLLC based on puncturing was proposed. In the Literature [8], a joint measurement based on the minimum reachable rate of eMBB and the optimal configuration requirement of URLLC was proposed to minimize the loss in a multi-service coexistence environment. The above work does not take into account that URLLC services are characterized by random and sporadic transmissions, which can result in idle channel resources.

To accommodate future power heterogeneous service traffic demands, diverse quality of service (QoS) demands, and enormous connection, non-traditional technologies such as non-orthogonal multiple access (NOMA) [9,10,11,12,13] are considered as core schemes beyond 5G and 6G networks [14,15]. The Literature [9] proposed an air-ground integrated C-NOMA heterogeneous power IoT (PIoT) network model. Each sub-channel of a high-altitude platform (HAP) was occupied by a user cluster and could be multiplexed by multiple unmanned aerial vehicles (UAVs) using NOMA. It delved into the task offloading and resource allocation issues. The Literature [12] proposed a 6G heterogeneous IoT C-NOMA system based on XAPS (including a HAP and multiple UAVs), where both the cluster terminals of the HAP and the terminals of the UAVs used NOMA to realize extensive and hotspot intensive coverage. In conclusion, the above work mainly targets a single type of user and uses NOMA without considering user service types and characteristics. In this paper, under the condition that the channel resources have been allocated to the URLLC service for transmission, when eMBB services request transmission, it is allowed to reuse their channel resources while ensuring the performance of URLLC services.

Due to limited communication resources, efficient resource allocation is a significant method to enhance system performance [16,17,18,19,20,21,22]. The Lliterature [16] proposed a heterogeneous network model for vehicular networking, which effectively improved the system energy efficiency and spectral efficiency through power control and sub-channel allocation. In the Literature [17], a space-air-ground power IoT architecture based on wireless transmission was proposed to achieve a balance between network reliability and running cost through task assignment and resource distribution. The resource allocation methods of the above work have a high computational complexity and the system performance still has room for improvement.

For settling the above issues, this paper firstly designs a NOMA-based model of a power IoT network for downlink transmission of URLLC and eMBB hybrid service scenarios. Second, considering the limited communication resources, a system throughput maximization problem with joint channel selection and power allocation is proposed. The problem involves several factors, such as channel resources and power, and is difficult to solve, so it is segmented into two problems that can be settled iteratively. The first subproblem is the channel selection problem of the users, the second subproblem is the power allocation problem for the users, and the approximate optimal results can be obtained by iterating the above two subproblems.

The major contributions of this paper can be summarized as follows:

(1) We develop a NOMA-based model of a power IoT network for downlink transmission of URLLC and eMBB combined service scenes. Among them, URLLC is typically characterized by low latency and high reliability, while eMBB focuses on transmitting large data blocks and high rate data and is mainly oriented to meet the service requirements of industrial and electric power industries. NOMA technology is used among users to achieve channel multiplexing. The model can be applied to different types of power business scenarios. Considering the limited communication resources, the problem of maximizing system throughput with joint channel selection and power allocation is proposed.
(2) Since the problem involves several factors such as channel resources and power, discrete variables and continuous variables exist simultaneously, and there is coupling between channel selection and power allocation, it is difficult to solve. Therefore, it is segmented into two problems that can be settled iteratively. In the first subproblem, the channel resources are classified and the channels are assigned based on the matching algorithm. In the second subproblem, the water injection power allocation is first performed for the channels to obtain the power of each channel, and then the fractional transmit power allocation method is employed for allocating the power to the users on the channels. By iterating the above two problems, the maximum system throughput is obtained.
(3) In this paper, a detailed theoretical analysis and abundant simulation experiments are carried out, and the proposed method is compared with other benchmark methods. The experimental results indicate that our method has superior performance in terms of system throughput and spectral efficiency.

## 2. System Model

**Figure 1:**
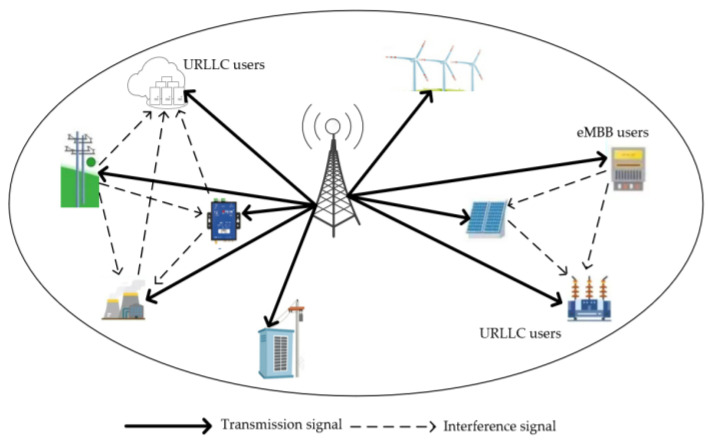
System Model.

Shown in Figure 1, this paper designs a NOMA-based power IoT network model for downlink transmission of URLLC and eMBB hybrid service scenarios, including one BS, $M=\left\{1,2,\ldots ,M\right\}$ URLLC users, and $N=\left\{1,2,\ldots ,N\right\}$ eMBB users. The frequency band resource with bandwidth W is divided into $K=\left\{1,2,\ldots ,K\right\}$ channels, of which L channels are private resources and K−L channels are common resources. The location of the BS is $U_{0}=\left(x_{u},y_{u}\right)$. The channel fading of the system follows Rayleigh fading.

The location of the mth URLLC user is denoted by $D_{m}$, $D_{m}=\left(x_{m},y_{m}\right)$. The distance between the URLLC user m and the BS is

(1) $$d_{m}=\sqrt{{\|D}_{m}-U_{0}{\|}^{2}}.$$

The distance between the eMBB user n and the BS is

(2) $$d_{n}=\sqrt{{\|D}_{n}-U_{0}{\|}^{2}}.$$

Let $P_{m,k}^{U}$ and $P_{n,k}$ be the assigned power of URLLC user m and eMBB user n in channel k. The sets of assigned power of URLLC users and eMBB users are defined as $P^{U}=\left\{P_{m,k}^{U},\forall m\in M,k\in K\right\}$ and $P=\left\{P_{n,k},\forall n\in N,k\in K\right\}$, respectively; the sets of channel selection between URLLC users, eMBB users, and BS are $A^{U}=\left\{a_{m,k}^{U},\forall m\in M,k\in K\right\}$ and $A=\left\{a_{n,k},\forall n\in N,k\in K\right\}$, respectively. When URLLC user m occupies the channel k, $a_{m,k}^{U}=1$, otherwise, $a_{m,k}^{U}=0$; when eMBB user n occupies the channel k, $a_{n,k}=1$, otherwise, $a_{n,k}=0$.

### 2.1. URLLC Communication Model

When there are too many transmission services in the common resources or the transmission conditions are too poor to achieve the transmission goal of URLLC services, private resources are allocated for URLLC services in priority. Moreover, the decoding of URLLC users is preferred in order to reduce the transmission delay, so that the signal-to-interference-noise ratio (SINR) of URLLC user m in channel k is expressed as

(3) $$\gamma_{m,k}^{U}=\frac{P_{m,k}^{U}h_{m,k}^{U}}{N_{0}+\sum_{i=1}^{N}a_{i,k}P_{i,k}h_{i,k}+\sum_{j=m+1}^{M}a_{j,k}^{U}P_{j,k}^{U}h_{j,k}^{U}},$$

where $h_{m,k}^{U}={\left|g_{m,k}^{U}\right|}^{2}d_{m}^{-\beta }$, β denotes the path loss index, ${\left|g_{m,k}^{U}\right|}^{2}$ is the small-scale fading gain of the URLLC user m, and $N_{0}$ is the additive white Gaussian noise.

Therefore, the throughput of URLLC user m on the channel k is

(4) $$R_{m,k}^{U}=\frac{W}{K}log_{2}\left(1+\gamma_{m,k}^{U}\right)-\frac{1}{ln2}\sqrt{\frac{C\left(\gamma_{m,k}^{U}\right)}{H}}Q^{-1}\left(\delta \right),$$

where $H=\frac{tW}{K}$ is the transmission block length of URLLC service, t is the transmission time, $C\left(\gamma_{m,k}^{U}\right)=1-\frac{1}{{\left(1+\gamma_{m,k}^{U}\right)}^{2}}$ denotes the channel dispersion, and $Q^{-1}\left(\delta \right)$ is the inverse function of $Q\left(\delta \right)=\frac{1}{\sqrt{2\pi }}\int_{\delta }^{\infty }e^{-\frac{t^{2}}{2}}dt$.

### 2.2. eMBB Communication Model

When the eMBB user decodes, there is only interference from other eMBB users on the channel k, so the SINR of the eMBB user n in the kth channel can be expressed as

(5) $$\gamma_{n,k}=\frac{P_{n,k}h_{n,k}}{N_{0}+\sum_{i=n+1}^{N}a_{i,k}P_{i,k}h_{i,k}},$$

where $h_{n,k}={\left|g_{n,k}\right|}^{2}d_{n}^{-\beta }$, ${\left|g_{n,k}\right|}^{2}$ is the small-scale fading gain of eMBB user n.

As a result, the throughput of eMBB user n on the channel k is

(6) $$R_{n,k}=\frac{W}{K}log_{2}\left(1+\gamma_{n,k}\right).$$

In summary, the total system throughput is expressed as

(7) $$R=\overset{K}{\underset{k=1}{\sum }}\left(\overset{M}{\underset{m=1}{\sum }}a_{m,k}^{U}R_{m,k}^{U}+\overset{N}{\underset{n=1}{\sum }}a_{n,k}R_{n,k}\right).$$

## 3. Problem Formulation

Maximizing the throughput of the power IoT network in a hybrid service scenario by co-designing channel selection and power allocation is the objective of this paper, which can be shown by:

(8) $$P0:\underset{A^{U},A,P^{U},P}{max}R$$

(9) $$C1:P_{n,k}\geq 0$$

(10) $$C2:P_{n,k}\leq p_{max}$$

(11) $$C3:\overset{K}{\underset{k=1}{\sum }}\overset{N}{\underset{n=1}{\sum }}a_{n,k}P_{n,k}+\overset{K}{\underset{k=1}{\sum }}\overset{M}{\underset{m=1}{\sum }}a_{m,k}^{U}P_{m,k}^{U}\leq P_{max}$$

(12) $$C4:P_{m,k}^{U}\geq 0$$

(13) $$C5:P_{m,k}^{U}\leq p_{max}^{u}$$

(14) $$C6:a_{n,k}\in \left\{0,1\right\}$$

(15) $$C7:a_{m,k}^{U}\in \left\{0,1\right\}$$

(16) $$C8:R\geq R_{min}.$$

Constraints C1–C2 are the power constraints for eMBB users, $p_{max}$ is the maximum allocated power for eMBB users, C3 is the power constraint for the coverage area of the BS, $P_{max}$ is the total transmit power of the BS, C4–C5 are the power constraints for URLLC users, $p_{max}^{u}$ is the maximum power sum for URLLC users, C6–C7 are the channel selection constraints for eMBB users and URLLC users, respectively, C8 is the QoS constraint, and $R_{min}$ is the minimum system throughput.

## 4. System Analysis

Since the problem involves multiple factors, discrete and continuous variables exist simultaneously, and there is coupling between channel selection and power allocation, which makes it difficult to solve, it is segmented into two problems that can be settled iteratively. In the first subproblem, the channel resources are classified and the channels are assigned based on the matching algorithm. In the second subproblem, the power allocation based on the water injection method is first performed for the channels to obtain the power of each channel, and then the fractional transmit power allocation method is employed for allocating the power to the users on the channels. By iterating the above two subproblems, the maximum system throughput is obtained.

### 4.1. Channel Selection

Based on the given power, the problem **P0** is transformed into an optimization problem on channel selection, which can be expressed as

(17) $$SP1:\underset{A^{U},A}{max}R$$

(18) $$C3:\overset{K}{\underset{k=1}{\sum }}\overset{N}{\underset{n=1}{\sum }}a_{n,k}P_{n,k}+\overset{K}{\underset{k=1}{\sum }}\overset{M}{\underset{m=1}{\sum }}a_{m,k}^{U}P_{m,k}^{U}\leq P_{max}$$

(19) $$C6:a_{n,k}\in \left\{0,1\right\}$$

(20) $$C7:a_{m,k}^{U}\in \left\{0,1\right\}$$

(21) $$C8:R\geq R_{min}.$$

We introduce the interruption probability as the reliability target of service transmission, and take URLLC users as an example. The interruption probability of URLLC user m is represented as

(22) $$p_{m,k}=Pr\left(R_{m,k}^{U}<r_{tv}\right)=\int_{0}^{\frac{1}{\gamma }\left(2^{r_{tv}}-1\right)}f\left(x\right)dx,$$

where $r_{tv}$ is the throughput threshold. When the throughput is less than the threshold value, the transmission is interrupted. $f\left(x\right)$ is the probability density function. Its expression is

(23) $$f\left(x\right)=\frac{x}{N_{0}}\exp\left(-\frac{x}{2N_{0}}\right),x\geq 0.$$

The interruption probability of URLLC user m is

(24) $$p_{m,k}=Pr\left(R_{m,k}^{U}<r_{tv}\right)=1-exp\left(-\frac{\frac{1}{\gamma }{\left(2^{r_{tv}}-1\right)}^{2}}{2N_{0}}\right).$$

Solve the throughput threshold:

(25) $$r_{tv}=log_{2}\left[1+\sqrt{2N_{0}\gamma^{2}ln\left(1-p_{m,k}\right)}\right].$$

When the URLLC user transmits, according to the information obtained by the BS, it will determine whether the throughput of the URLLC user in the common resource can be greater than the throughput threshold. For the URLLC user who can meet the throughput threshold, its transmission channel will be selected from the common channel resources, and for the URLLC user who cannot reach the throughput threshold, the private channel resources will be assigned for transmission first. The part of the user’s required resources that exceeds the number of private resources will be selected from the common resources, so that the users’ channel selection is completed.

The problem of channel selection is transformed into a one-to-one matching game using matching game theory, and a channel is set to accommodate at most one URLLC user and one eMBB user. The channels and the users are two sets of participators. In order to maximize the system throughput, the users are matched with the channels to find a solution. It is defined as a matching mapping between the set of users M/N and the set of channels K. Taking eMBB users as an example, under this model, each user only cares about the throughput of the occupied channel, thus obtaining the utility function.

For each channel k, it is concerned with the reachable rate of all users occupying channel k. So the utility function of matching channel k is obtained.

Each user can be matched to only one channel. When a user matches with channel $k_{1}$ with higher throughput than with channel $k_{2}$, the user prefers channel $k_{1}$ to channel $k_{2}$. In exchange matching, two users exchange the channels they match when the other matches remain unchanged. If the utility of one or more participants increases while the utility of the other participants does not decrease, then this exchange matching is called a blocking pair. For a blocking pair, each user hopes to match with other participants, instead of maintaining matches with the present matching participant. It is said to be steady if there is no blocking pair in the match.

According to the above-mentioned definition, the channel selection algorithm based on matching is proposed. As shown in Algorithm 1, first, an initial matching is given in which the users and channels are randomly matched. Next, two different channels are randomly selected for exchange matching. Then the utility is calculated. In case there exists a blocking pair, the exchange operation is carried out. The exchange matching goes on until there are no blocking pairs. At last, steady matching is realized.

The system rate increases after each switching operation. In a limited number of switching operations, a stable match can be found. After each exchange, R increases.
**Algorithm 1** Channel selection algorithm based on matching1. Initialize preference lists $PL_{n}^{E}$, $PL_{k}^{C}$ for eMBB users and channels2. Set the matching set $MC^{E}=\emptyset$, op=1;3. while op==1 do4. Set op=0;5.  for each eMBB user n do6.   if $PL_{n,k}^{E}\neq \emptyset$
7.    Select the optimal channel $c_{k}$ in the preference list $PL_{n}^{E}$ as $MC^{E}\left(u_{n}\right)$;8.   if the number of participants of $MC^{E}\left(u_{n}\right)==$ maximum capacity of the channel9.    Select the worst matching user $u_{n}^{\prime }$ in $MC^{E}\left(c_{k}\right)$;10.          if $u_{n}$ is better than $PL_{n}^{E}\left(u_{n}^{\prime }\right)$
11.    Swap matching $u_{n}$ and $u_{n}^{\prime }$ in $MC^{E}\left(c_{k}\right)$;12.          else13.     $PL_{n}^{E}=PL_{n}^{E}\backslash c_{k}$;14.          end if15.          Set op=1
16.          end if17.          $MC^{E}=MC^{E}\cup \left(u_{n},c_{k}\right)$
18.          end if19.   end for20. end while21. Repeat steps (1)–(20) to obtain the matching result $MC^{U}$ for the URLLC users.22. Output matching results $MC^{U}$ and $MC^{E}$.

### 4.2. Power Optimization

Based on the obtained channel selection results, the users’ allocated power is optimized. The problem can be formulated as

(26) $$SP2:\underset{P^{U},P}{max\;R}$$

(27) $$C1:P_{n,k}\geq 0$$

(28) $$C2:P_{n,k}\leq p_{max}$$

(29) $$C3:\overset{K}{\underset{k=1}{\sum }}\overset{N}{\underset{n=1}{\sum }}a_{n,k}P_{n,k}+\overset{K}{\underset{k=1}{\sum }}\overset{M}{\underset{m=1}{\sum }}a_{m,k}^{U}P_{m,k}^{U}\leq P_{max}$$

(30) $$C4:P_{m,k}^{U}\geq 0$$

(31) $$C5:P_{m,k}^{U}\leq p_{max}^{u}$$

(32) $$C8:R\geq R_{min}.$$

Furthermore, a power allocation algorithm based on the water injection method is designed. First, using the water injection method to allocate channel power, the inter-channel power allocation problem is specifically described as

(33) $$SP2^{\prime }:\underset{P_{k}}{max}\frac{W}{K}\overset{K}{\underset{k=1}{\sum }}log_{2}\left(1+p_{k}H_{k}\right)$$

(34) $$C1^{\prime }:p_{k}\geq 0$$

(35) $$C2^{\prime }:p_{k}\leq p_{max}+p_{max}^{u}$$

(36) $$C3^{\prime }:\overset{K}{\underset{k=1}{\sum }}p_{k}\leq P_{max}$$

(37) $$C9^{\prime }:\overset{K}{\underset{k=1}{\sum }}R_{k}\geq R_{min}.$$

where $H_{k}$ is the equivalent channel gain of channel k, and $p_{k}\in P_{K}$ is the total power allocated on the channel k.

Next, construct the Lagrangian function:

(38) $$L=\frac{W}{K}\sum_{k=1}^{K}log_{2}\left(1+p_{k}H_{k}\right)-\sum_{k=1}^{K}\mu_{k}p_{k}+\sum_{k=1}^{K}\lambda_{k}\left(p_{k}-p_{max}-p_{max}^{u}\right)+\;\gamma \left(\sum_{k=1}^{K}p_{k}-P_{max}\right)+\varpi \left(R_{min}-\sum_{k=1}^{K}R_{k}\right),$$

where $\mu_{k}$, $\lambda_{k},\gamma$, ϖ are the Lagrange multipliers.

In Equation (38), the partial derivative of $p_{k}$ is obtained as

(39) $$\frac{\partial L}{\partial p_{k}}=\frac{W}{Kln2}\frac{H_{k}}{1+p_{k}H_{k}}-\mu_{k}-\lambda_{k}+\gamma =0.$$

From Equation (39), we get

(40) $$p_{k}=\frac{W}{\gamma Kln2}-\frac{1}{H_{k}}.$$

Let $\theta =W/\left(\gamma Kln2\right)$, and the above equation can be expressed as

(41) $$p_{k}=\theta -\frac{1}{H_{k}},$$

where θ is the water level at the time of water injection.

The power $p_{k}$ of each channel is obtained, and then the fractional transmit power allocation (FTPA) [23] method is employed for allocating the power to the users on the channels. The allocated power of URLLC user m on the channel k is formulated as

(42) $$p_{m,k}=\frac{p_{k}}{\sum_{j\in T_{k}}h_{j,k}^{-2\alpha_{F}}}h_{m,k}^{-2\alpha_{F}},$$

where $T_{k}$ denotes the set of users on channel k, and $\alpha_{F}$ denotes the fading factor ($0\leq \alpha_{F}\leq 1$). When $\alpha_{F}=0$, the power is equally distributed among users, and as $\alpha_{F}$ increases, users with small channel gain will be allocated more power.

Similarly, the allocated power for eMBB user n is formulated as

(43) $$p_{n,k}=p_{k}-p_{m,k}.$$

As shown in Algorithm 2, the overall algorithm of power allocation is as follows.
**Algorithm 2** Power allocation algorithm based on water injection method 1. Set the water level for the initial water injection, and let $\theta =\frac{1}{K}\left(P_{max}+\overset{K}{\underset{k=1}{\sum }}\frac{1}{H_{k}}\right)$;2. The channel state values of each channel are arranged in ascending order from smallest to largest, and the power values are also arranged in ascending order at this time.3. Use Equation (41) to calculate the power allocated to each channel.4. If the power assigned to a channel is less than 0, then set its value to 0 and eliminate it in the next iteration; if the power assigned to a user is greater than $p_{max}+p_{max}^{u}$, then set its value to $p_{max}+p_{max}^{u}$ and return to step 2 to finally obtain the transmit power of all channels.5. The allocated power of users in each channel is calculated by Equations (42) and (43), and if the allocated power is greater than its maximum value, the maximum value is taken to finally complete the power allocation.

The optimization results of channel selection and power allocation are obtained by iterating Algorithm 1 and Algorithm 2 until convergence.

### 4.3. Performance Analysis

Since the preference profiles are finite and shorten each time they are rejected by the agent, the loop of Algorithm 1 terminates once all preference profiles are associated to be empty. Similarly, the number of channels is finite, and convergence is reached quickly in Algorithm 2. Therefore, our method has preferable convergence. In the matching algorithm, the preference list length is K and the complexity of the user’s preference profile is $O\left(Klog\left(K\right)\right)$. The loop terminates when each channel is assigned to a user or its preference profiles is empty. At each rejection, the users then create a preference list. Each user can be rejected at most K times, and the computational complexity is $O\left(\left(M+N\right)Klog\left(K\right)\right)$. In the power allocation process, the complexity of the water injection method is $O\left(iK\right)$, where i denotes the number of iterations. Overall, the computational complexity of the proposed method is $O\left(I\left(\left(M+N\right)Klog\left(K\right)+iK\right)\right)$, and I is the number of iterations of the two algorithms. Compared with the exhaustive method, the complexity is greatly reduced.

## 5. Experimental Analysis

This section verifies the performance of the proposed method through abundant simulation experiments. The coverage area of the BS is 500 m × 500 m, the total system bandwidth is 1 MHz, and the number of channel resources and private channel resources are 8 and 2, respectively. The number of common channel resources is 6, and the path loss factor is 2. Table 1 is a detailed list of simulation parameters.

**Figure 2:**
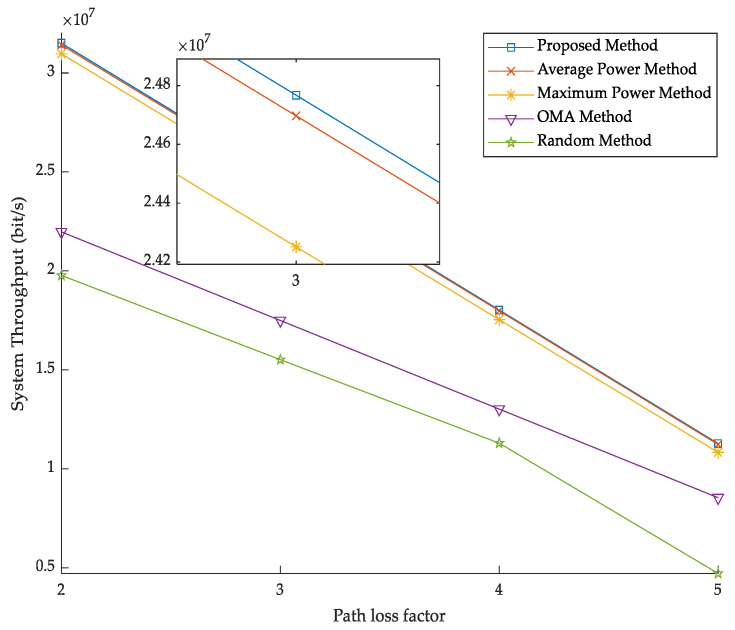
Total system throughput versus path loss factor.

Figure 2 investigates the effect of the path loss factor on system throughput under different methods, including the average power method, maximum power method, OMA method, random method, and the proposed method. Figure 2 shows that the system throughput is decreasing as path loss factor increases because the larger the path loss, the smaller the channel gain and the lower the throughput. The proposed method is superior in terms of throughput compared to other methods because the proposed method can make full use of system resources by optimizing channel selection and transmission power strategies, thus improving system throughput. Compared with the average power method and the maximum power methods, the performance of the proposed method is improved by 0.4% and 2%, respectively. This is because the average power method and the maximum power method cannot dynamically adjust power resources. Compared with the OMA method, the performance of the proposed method is improved by 43%, which is due to the low channel utilization of OMA method, resulting in poor performance. The random method does not consider the optimization of channel and power, and its performance is the lowest.

**Figure 3:**
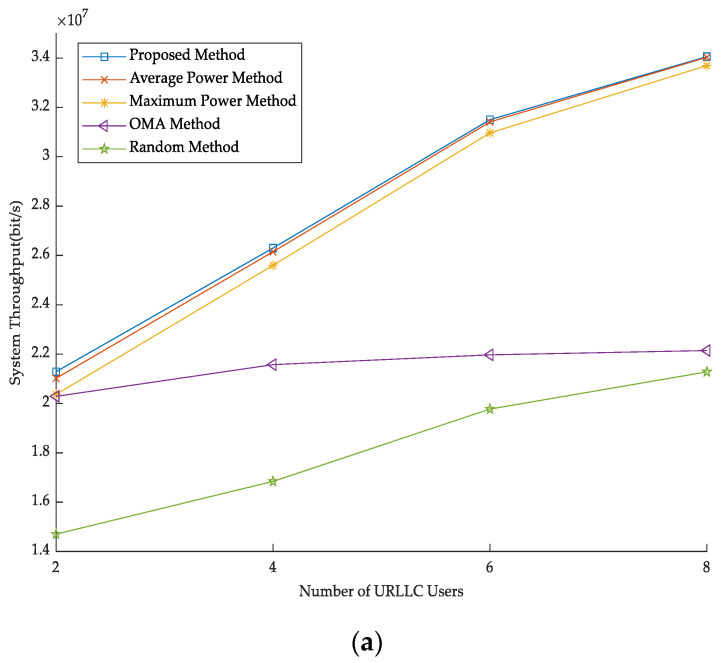

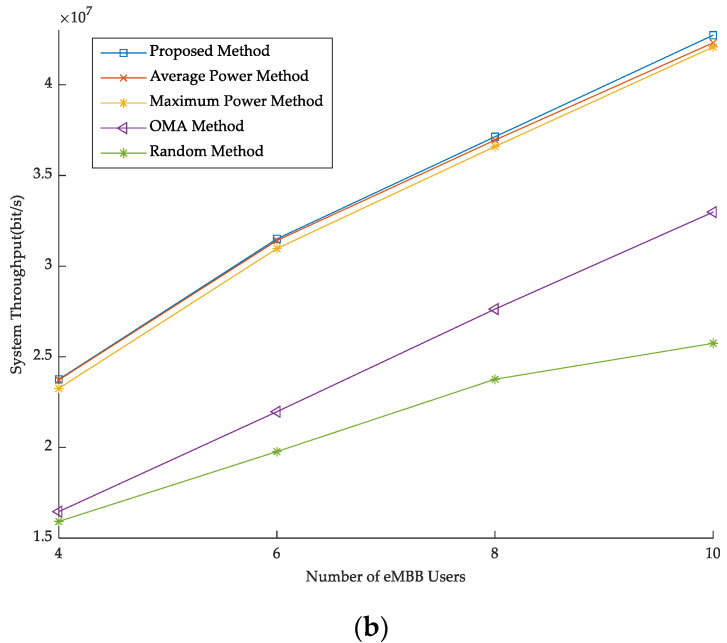
The number of users versus throughput. (**a**) The number of URLLC users versus throughput. (**b**) The number of eMBB users versus throughput.

Throughput is greatly affected by the number of users under different methods. Figure 3 shows the number of users with throughput. Along with the increase of users, the system throughput is also increasing. According to Figure 3, compared with the OMA method, our approach makes the most of channel resources and improves channel utilization. The throughput of the proposed method is also superior to that of several other methods. This is because the proposed method takes into account the channel and power and has better performance, which reflects the effectiveness of the proposed method.

**Figure 4:**
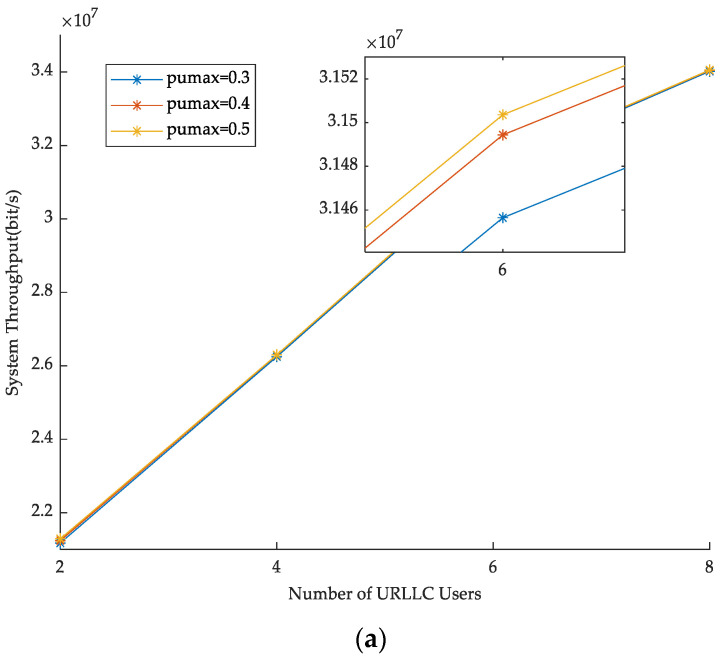

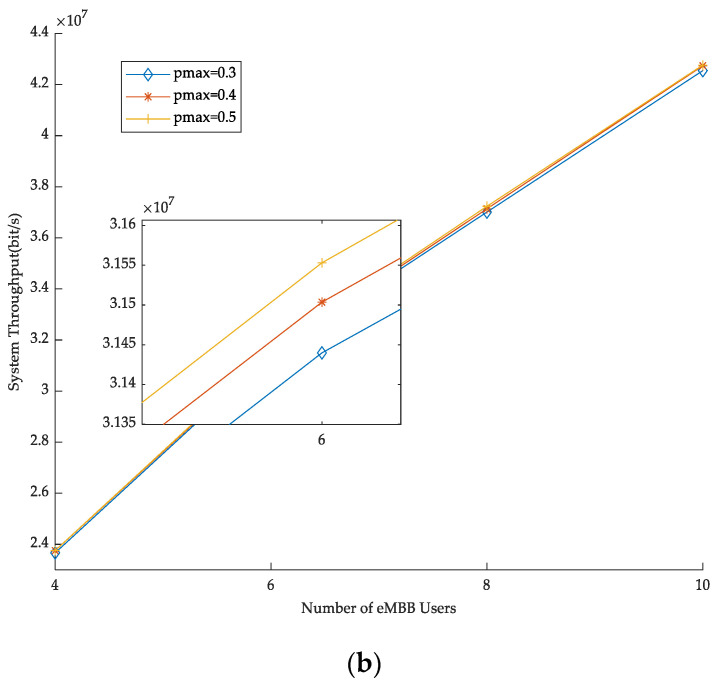
Maximum allocated power of users versus throughput. (**a**) Maximum power allocated by URLLC users versus throughput. (**b**) Maximum power allocated by eMBB users versus throughput.

In Figure 4, the BS power $P_{max}$ is fixed. By changing the number of URLLC and eMBB users, the effect of the maximum allocated power of users on throughput is observed. From the figure, it can be seen that for the same maximum allocated power, the more users, the higher the system throughput. When the maximum allocated power of URLLC users and eMBB users increases, system throughput also improves. The reason is that the greater the allocated power, the larger the overall system growth rate, resulting in higher system throughput.

**Figure 5:**
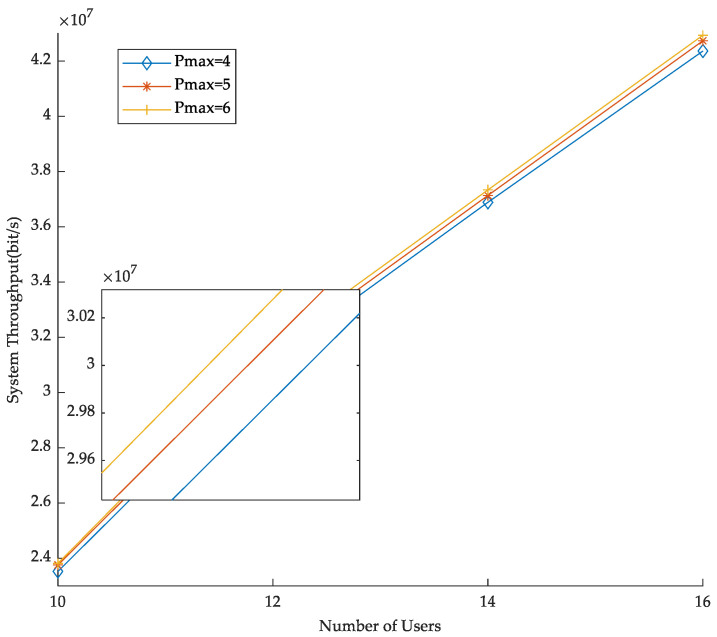
BS power versus throughput.

In Figure 5, the maximum allocated power of URLLC and eMBB users is fixed. By changing the number of users, the effect of BS power on throughput is observed. Specifically, the throughput improves with the number of users for the same BS transmit power. Additionally, for systems with the same number of users, the higher the BS transmit power, the more power the users are allocated under the maximum allocated power constraint, resulting in an increase in transmission rate and system throughput.

**Figure 6:**
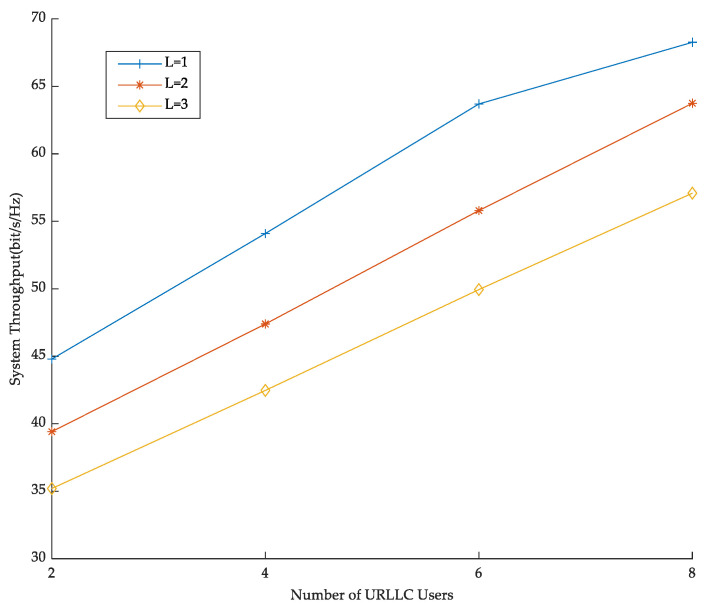
The number of private channels versus spectrum efficiency.

Figure 6 shows the effect of the number of private channels on spectrum efficiency. In Figure 6, the maximum allocated power of users and the BS transmit power are fixed. By changing the number of URLLC users, the change of the system performance is observed. As you can see, spectral efficiency improves with the increase of users for the same number of private channels. The reason is that an increase in the number of users causes the transmission rate to increase, leading to a growth in spectral efficiency. Meanwhile, for the system with the same number of URLLC users, the more private channel resources, the lower the spectral efficiency, which is due to the fact that the fewer private channels, the higher the channel utilization and the higher the spectral efficiency, reflecting the superiority of NOMA technology.

## 6. Conclusions

In this paper, a downlink power IoT network model based on NOMA for URLLC and eMBB hybrid service scenarios is proposed, and the problem of system throughput maximization is formulated through joint optimization of channel selection and power allocation. Because of the coupling, the problem is decomposed into two subproblems, and we design a channel selection algorithm based on matching and a power allocation algorithm based on the water injection method to maximize the system throughput. Theoretical analysis and simulation results show that the proposed method has superior performance in system throughput and spectrum efficiency in power hybrid service scenarios. In the future, we will consider integrating the space-air-ground heterogeneous network into the power network and explore more complex application scenarios.

## Figures and Tables

**Table 1:** Simulation parameters.

Parameters	Values
Number of URLLC users M	6
Number of channels K	8
Number of eMBB users N	6
Path loss factor β	2
Additive Gaussian white noise $N_{0}$	−174 dBm/Hz
URLLC user maximum allocated power $p_{max}^{u}$	0.5 W
eMBB user maximum allocated power $p_{max}$	0.4 W
BS transmitting power $P_{max}$	5 W
Fading factor $\alpha_{F}$	0.2
Time delay constraint *t*	1 ms

## Data Availability

Not applicable.
